# A Combinatorial Approach to Regenerate the Periodontal Ligament and Cementum in a Nondental Microenvironment

**DOI:** 10.1155/2023/1277760

**Published:** 2023-07-28

**Authors:** Yongwen Guo, Mengting He, Peiqi Wang, Ding Bai, Jeong-Hui Park, Khandmaa Dashnyam, Jung-Hwan Lee, Olivier Huck, Nadia Benkirane-Jessel, Hae-Won Kim, Murugan Ramalingam

**Affiliations:** ^1^State Key Laboratory of Oral Diseases, National Clinical Research Center for Oral Diseases, Department of Orthodontics, West China Hospital of Stomatology, Sichuan University, Chengdu 610041, China; ^2^Lanzhou Stomatological Hospital, Lanzhou 730031, China; ^3^Institute of Tissue Regeneration Engineering, Dankook University, Cheonan 31116, Republic of Korea; ^4^Department of Nanobiomedical Science, BK21 NBM Global Research Center for Regenerative Medicine, Dankook University, Cheonan 31116, Republic of Korea; ^5^Mechanobiology Dental Medicine Research Center, Dankook University, Cheonan 31116, Republic of Korea; ^6^INSERM UMR 1260, Regenerative Nanomedicine, University of Strasbourg, Strasbourg 67084, France; ^7^IKERBASQUE, Basque Foundation for Science, Bilbao 48013, Spain; ^8^Joint Research Laboratory on Advanced Pharma Development Initiative, A Joined Venture of TECNALIA and School of Pharmacy, University of the Basque Country (UPV/EHU), Vitoria-Gasteiz 01006, Spain; ^9^Biomedical Research Networking Center in Bioengineering, Biomaterials and Nanomedicine (CIBER-BBN), Carlos III Health Institute, Madrid 28029, Spain; ^10^Bioaraba Health Research Institute, Jose Atxotegi, s/n, Vitoria-Gasteiz 01009, Spain; ^11^School of Basic Medical Sciences, Chengdu University, Chengdu 610106, China; ^12^School of Basic Medical Sciences, Binzhou Medical University, Yantai 264003, China; ^13^Department of Metallurgical and Materials Engineering, Atilim University, Ankara 06830, Turkey; ^14^Institute of Precision Medicine, Furtwangen University, 78054 Villingen-Schwenningen, Schwarzwald, Germany

## Abstract

While treated dentin matrix (TDM) has been used for regeneration of dental tissues, the quality and quantity of regenerated periodontal tissue structure are suboptimal. The present study was undertaken to test whether the combined use of the TDM with dental follicle cells (DFCs) and Hertwig's epithelial root sheath (HERS) cells enhances the regeneration of periodontal structures in a nondental microenvironment. TDMs were fabricated from 3-month-old Sprague–Dawley (SD) rats. DFCs and HERS cells were isolated from postnatal 7-day SD rats. Purified DFCs and HERS cells, both in combination or alone, were seeded and cultured on TDM *in vitro* and characterized. The cell-seeded TDMs were subsequently implanted into a 3-month-old rat greater omentum for 6 weeks, and further histological evaluation was performed. The results showed that cells grew well on the surface of TDMs, and mineralized nodules could be seen, especially in the HERS + DFCs group. After transplantation in rat omentum, periodontal ligament-like fibers and cementum-like structures were observed around the TDM in 1/3 of the samples in both the HERS group and the DFCs group and in 2/3 of the samples in the HERS + DFCs group, while almost no attached tissue formation was found in the TDM only group. The formed cementum width and the periodontal ligament length were significantly larger in the HERS + DFCs group. The periodontal ligament-like fibers in the HERS + DFCs group were orderly arranged and attached to the cementum-like tissues, which resembled the cementum-periodontal structure. Therefore, the combined use of DFCs, TDM, and HERS cells may be a promising strategy for the regeneration of the periodontal structures, especially in the nondental microenvironment.

## 1. Introduction

The tooth and its surrounding tissues form a tooth-periodontal ligament-alveolar bone structure, which becomes the exceptional histological basis for supporting the tooth as it performs its physiological functions [1]. Lesions to this structure, such as periodontitis, will weaken the normal functions of the teeth, affecting people's quality of life. Therefore, repairing the defective periodontal tissues and reconstructing their normal structures and functions are the ultimate goals of periodontal therapy. However, due to the special structure of the periodontal tissue, effective means and measures to repair and reconstruct periodontal tissues are still lacking [2, 3].

The rapid development of regenerative medicine in recent years has made periodontal tissue engineering an alternative for the repair of periodontal defects [4]. To achieve the regeneration of periodontium, there are many critical factors including but not limited to the selections of biological materials, seed cells, and the establishment of an inductive microenvironment. The periodontal regeneration must be achieved on the basis of its developmental principles [5, 6]. It has been well acknowledged that the formation of root dentin and its interactions with dental follicle cells (DFCs) and Hertwig's epithelial root sheath (HERS) are critical to the development of the periodontal tissues. Root formation begins with the formation of HERS, which exists as a boundary between the dental follicle and papilla [7]. Its inner surface induces the differentiation of odontoblast. When the outermost layer of dentin begins to calcify, HERS detaches from its surface and disintegrates. As the HERS disintegrates, DFCs move through the fenestrated HERS and attach to the newly formed dentin. With the induction of root dentin and HERS cells, DFCs differentiate into various kinds of cells that will form periodontal tissues in the future. The interactions among HERS, dentin matrix, and dental follicle direct the root and periodontal tissue formation. Therefore, one of our research goals was to find a suitable scaffold material with biological activity that can simulate dentin and to enhance periodontal regeneration through the interactions among HERS, DFCs, and the scaffold materials under the induction of the local microenvironment.

The successful use of acellular matrix scaffolds in tissue engineering has inspired the development of the treated dentin matrix (TDM), a decellularized matrix scaffold derived from dentin [8, 9]. Studies have shown that the TDM possesses natural microstructures, good biocompatibility, and bioactivity, which could release a variety of essential cytokines involved in tooth tissue formation [8, 10]. TDM could potentially provide an inductive microenvironment and is likely an excellent scaffold material for periodontal regeneration. When TDM was combined with DFCs and transplanted into fresh extraction alveolar sockets, cementum-periodontal ligament-like structures can be regenerated. However, a similar result was not seen on other graft sites, such as the healed alveolar bone, subcutaneous tissues, and the greater omentum, where the regeneration of cementum and periodontal ligament were compromised [8, 11, 12]. The above phenomena suggested that the periodontal regeneration by the combined use of only DFCs and TDM was microenvironment dependent. The cell composition of the microenvironment varies according to the sites of transplantation. In fresh extraction alveolar sockets, there may be residual dental epithelial rests of Malassez cells and various dental mesenchymal cells. Based on the developmental principle, the formation of periodontal tissues is the result of epithelial-mesenchymal interaction. These residual epithelial and mesenchymal cell components in the fresh extraction alveolar sockets could be directly or indirectly involved in the formation of periodontal tissue [13, 14]. However, the dental epithelial and mesenchymal components are absent in the nondental microenvironments, such as the healed alveolar bone, the subcutaneous tissues, or the omentum [15]. Therefore, transplantation of the suitable seed cells to the nondental microenvironments is a promising approach for periodontal regeneration.

Studies have confirmed the role of HERS and DFCs in periodontal development and their potential for periodontal regeneration [16–18]. HERS cells are essential in cementum formation and are able to induce the differentiation of DFCs to form periodontal tissues [19, 20]. Previous studies have either combined only DFCs and TDM or DFCs and HERS cells. On the one hand, the combination of the three components for periodontal regeneration remains unclear; on the other hand, whether regeneration could be achieved without limitation to the transplantation microenvironment still needs to be further explored. Therefore, the present study was designed to test whether the combined use of TDM with DFCs and HERS cells enhances the regeneration of periodontal structures in a nondental microenvironment. HERS cells and DFCs obtained from postnatal 7-day SD rats, either in combination or alone, were combined with rat TDMs *in vitro* and then transplanted into the 3-month-old SD rat omentum for 6 weeks, and further histological evaluation was performed. The carrying out of this study would provide strategies for periodontal tissue regeneration, especially in a nondental microenvironment.

## 2. Materials and Methods

**Figure 1:**
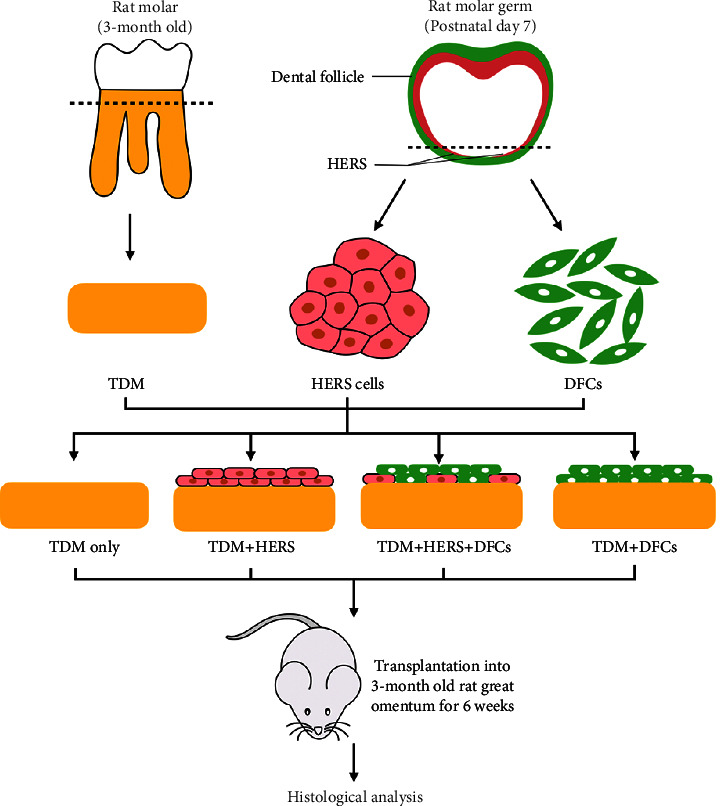
Schematic diagram of the research strategy. TDMs were fabricated from the 3-month-old rat roots of molars, while HERS cells and DFCs were obtained from the molar germs of postnatal day-7 Sprague–Dawley rats. HERS cells and DFCs were seeded on TDMs individually and in combination, cultured *in vitro,* and then these complexes were transplanted into the rat greater omentum for 6 weeks for further histological analyses.

The schematic diagram of the overall research strategy for the present study is illustrated in Figure 1.

### 2.1. Cell Isolation, Culture, and Identification

DFCs and HERS cells were isolated from molar germs of postnatal day 7 Sprague–Dawley rats referring to a modified method as previously described [21, 22]. HERS cells were cultured in epithelial cell medium (EpiCM) (Sciencell, San Diego, CA, USA), and DFCs were cultured in *α*-MEM medium (Gibco, Grand Island, NY, USA), supplemented with 10% fetal bovine serum (Gibco) and 1% penicillin/streptomycin (P/S) (Beyotime, Shanghai, China). Cells were incubated at 37°C in a humidified environment with 5% of CO_2_, and the medium was changed every two days. The cells were then identified by immunofluorescence staining of CK-14 and vimentin. Briefly, the prepared cell slides were fixed with 4% paraformaldehyde (Biosharp, Hefei, China) for 20 min, followed by permeabilization with 0.5% Triton-X-100 (Biofroxx, Einhausen, Germany) on ice, blocking with 5% bovine serum albumin (Biofroxx, Einhausen, Germany) for 30 min at 37°C and then incubation with anti-CK-14 (1 : 200) (Affinity, USA) or antivimentin (1 : 200) (Affinity) overnight at 4°C. The cell slides were then incubated with biotin-labeled goat antirabbit IgG (1 : 10000) (SAB, Maryland, USA) for 1 h at 37°C and with 4′,6-diamidino-2-phenylindole (DAPI) (Solarbio, Beijing, China) for 3 min. The cell slides were observed and photographed under a fluorescence microscope (Leica, Germany). The mean gray values of the fluorescence were further analyzed with ImageJ (NIH, USA).

### 2.2. Fabrication of TDM

**Figure 2:**
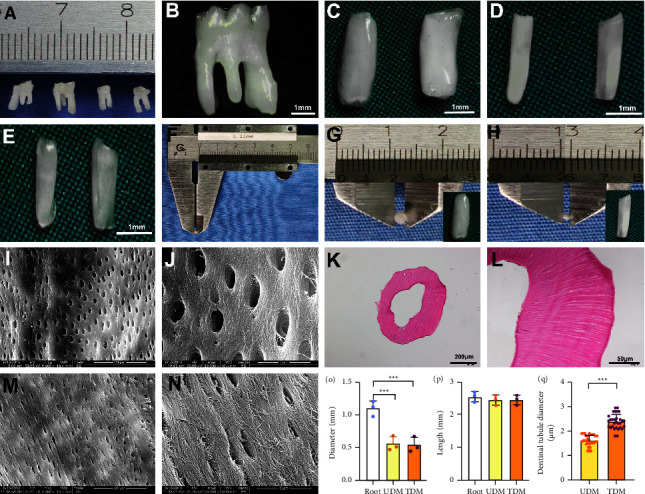
Fabrication and evaluation of the rat treated with dentin matrix (TDM). (a, b) Molars harvested from 3-month-old rats; (c) single root dissected; (d) the untreated dentin matrix (UDM) was obtained by removing the outer cementum, periodontal ligament, and the inner dental pulp; (e) after ultrasonic cleaning and gradient demineralization by EDTA solution; size measurement of the root (f, g) and the fabricated TDM (h); H&E staining showed complete removal of the outer periodontal ligament and cementum, as well as the cell components (k, l); SEM examination revealed that TDM (i, j) showed better exposure of dentinal tubules compared with the UDM (m, n), and the diameters of the TDM dentinal tubules were significantly larger than the UDM (q). (o, p) The comparison of the diameter and the length of the root, UDM, and TDM (^*∗∗∗*^*p* < 0.001).

TDM was fabricated with reference to a modified method described previously [8]. Briefly, first molars were harvested from five 3-month-old SD rats (Figure 2(a)). The periodontium was completely scraped away, and the roots were divided into singles (Figures 2(b) and 2(c)). The untreated dentin matrix (UDM) was obtained by removing the external periodontal ligament and cementum completely. The inner dental pulp was also removed (Figure 2(d)). After ultrasonic cleaning and gradient demineralization, TDMs were obtained (Figure 2(e)). The size of the root, UDM, and TDM was measured, respectively (Figures 2(e)–2(h)). The fabricated TDMs were cylindrical in shape, with an approximate length of 2.5 mm and a diameter of 0.5 mm (Figures 2(e), 2(h), 2(o), and 2(p)). The TDM was then treated with a sterilized PBS solution containing 100 units/mL of penicillin/streptomycin for 72 h and subsequently stored in *α*-MEM containing 50 units/mL P/S at 4°C for later use. The exposure of the dentinal tubules and the morphological surface structure of UDM and TDM were observed by scanning electron microscopy (SEM) (Thermo Fisher Scientific, Waltham, MA, USA). The dentinal tubule diameters were measured at 5000x magnification of SEM. 30 dentinal tubule diameters were measured on each specimen. The TDM was also stained with hematoxylin and eosin (H&E) (Beyotime, Shanghai, China) after sectioning.

### 2.3. Cell Seeding and SEM Examination

The prepared TDMs were first placed in 48-well plates, and cell suspensions were prepared for cell seeding. The TDMs in groups 1 (HERS) and 2 (HERS + DFCs) were seeded with HERS cell suspensions at an initial amount of 5 × 10^5^ according to Table 1. The unattached HERS cells were rinsed and removed after 24 h and cultured *in vitro* for another 24 h. Afterwards, DFCs were seeded with an initial amount of 5 × 10^5^ in group 2 and group 3 according to Table 1. The unattached DFCs were also rinsed and removed after 24 h and cultured for another 24 h. The culture medium for all groups was the combination of EpiCM and *α*-MEM with a mixing ratio of 1 : 1. The sequence of cell seeding of HERS and DFCs was to simulate the adjacent relationships of dentin, HERS, and dental follicle during the root development (Figure 1). Group 4 was seeded without cells and served as a blank control. For each group, 3 samples were prepared. Cell growth and cell morphology on TDMs were detected by SEM after *in vitro* culture.

### 2.4. *In Vivo* Transplantation and Histological Analysis

For each group, 3 replicates of TDM complexes seeded with or without cells (Table 1) were transplanted into a 3-month-old rat greater omentum and harvested after six weeks under deep anaesthesia (35 mg/kg pentobarbital, intraperitoneal injection). Samples were fixed with 4% paraformaldehyde solution at 4°C for 7 days and then demineralized with 10% ethylenediaminetetraacetic acid (EDTA) solution (PH = 8) at 37°C for 4 weeks, with the change of EDTA solution twice a week. Afterwards, the samples were rinsed with running water for 2 days to eliminate the EDTA. All samples were then embedded in paraffin and sectioned for HE and Masson's trichrome staining. Immunohistochemical staining using BSP antibody (Abcam, Cambridge, UK) and periostin antibody (Abcam) at 1 : 200 dilution was also performed. Histological images were taken under the microscope (Olympus, Tokyo, Japan). ImageJ 1.8.0 was used to measure the cementum width and periodontal length.

### 2.5. Statistical Analysis

GraphPad Prism 8.0 software was used to perform statistical analysis. The data were presented as the mean ± SD from at least three independent measurements. A one-way analysis of variance (ANOVA) followed by a post hoc Tukey's test were performed for the the statistical comparisons. *p* values less than 0.05 (^*∗*^*p* < 0.05, ^*∗∗*^*p* < 0.01, ^*∗∗∗*^*p* < 0.001, and ^*∗∗∗∗*^*p* < 0.0001) were considered statistically significant.

## 3. Results

### 3.1. Cell Culture and Identification

**Figure 3:**
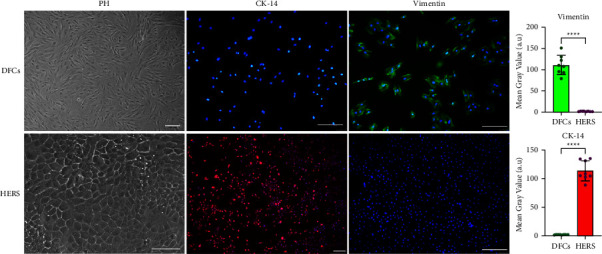
Purified DFCs and HERS cells were observed under a phase-contrast microscope (PH) and identified by immunofluorescence staining. HERS cells were positive for CK-14 (red fluorescence) but negative for vimentin (green fluorescence); DFCs showed the opposite staining of CK-14 and vimentin to HERS cells (^*∗∗∗∗*^*p* < 0.0001). The scale bars represent 200 *μ*m.

The purified DFCs and HERS cells were obtained after two rounds of differential trypsinization. HERS cells exhibited a typical epithelial cell-like morphology, and DFCs exhibited a typical fibroblast-like morphology. HERS cells were positively stained in red for the cytokeratin 14 (CK-14) but negatively stained green for the vimentin, while DFCs showed the opposite pattern to HERS (Figure 3).

### 3.2. The Fabricated TDMs Met the Experimental Requirements

H&E staining and SME observation (Figures 2(i)–2(l)) showed that the surface of the prepared TDM was free of stain, cementum, and periodontal ligament as well as the cell components. The peritubular and intertubular dentin fiber bundles became loose, and the dentinal tubules were completely exposed (Figures 2(k) and 2(l)). Compared with the UDM, the diameters of the TDM dentinal tubules were significantly larger (Figures 2(m)–2(q)). The above test results confirmed that the fabricated TDMs met the requirements for our study.

### 3.3. Effects of TDM on the Proliferation and Morphology of DFCs and HERS Cells

**Figure 4:**
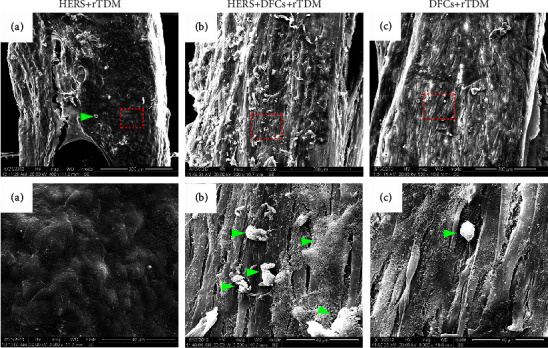
SEM examination showed the morphology of HERS cells and DFCs growing on TDM. (a) HERS cells grew in layers with a pebble-like morphology on the surface of TDM, and tight intercellular connections and abundant extracellular matrix could be seen; (b) HERS cells + DFCs: DFCs grew densely, spread over the surface of TDM in a membrane-like manner and arranged in bundles. There were fiber bundle-like synapses between the DFCs, while HERS cells were not visible underneath. Numerous mineralized nodules on the surface could be found (indicated by green arrows); (c) DFCs arranged into bundles and covered the surface of TDM in a membrane-like manner; a small amount of mineralized nodules were found on the surface (indicated by green arrows). The red frame indicated the areas for zooming in (a), (b), and (c).

SEM evaluations showed the cells in each group grew well on the surface of TDMs (Figure 4). In HERS cell group (Figure 4 (A, a)), cells grew densely on the surface of the TDMs in layers of pebble-like morphology with abundant extracellular matrix. In the HERS + DFCs group (Figure 4 (B, b)), cells grew densely in a membrane-like manner and were arranged in bundles that spread over the surface of the TDM. The HERS cells were covered by the DFCs and not visible. Like the HERS + DFCs group, the DFC group (Figure 4 (C, c)) also showed favorable cell growth with similar cell morphology and arrangement. More importantly, different amounts of mineralized nodules were found on the surface of TDMs among groups. The HERS + DFCs group showed the most amount of mineralized nodules, while the HERS group showed the least.

### 3.4. Abundant Cementum-Like and Periodontal Ligament-Like Tissues Formed by the Combined Use of TDM, DFCs, and HERS after *In Vivo* Transplantation

**Figure 5:**
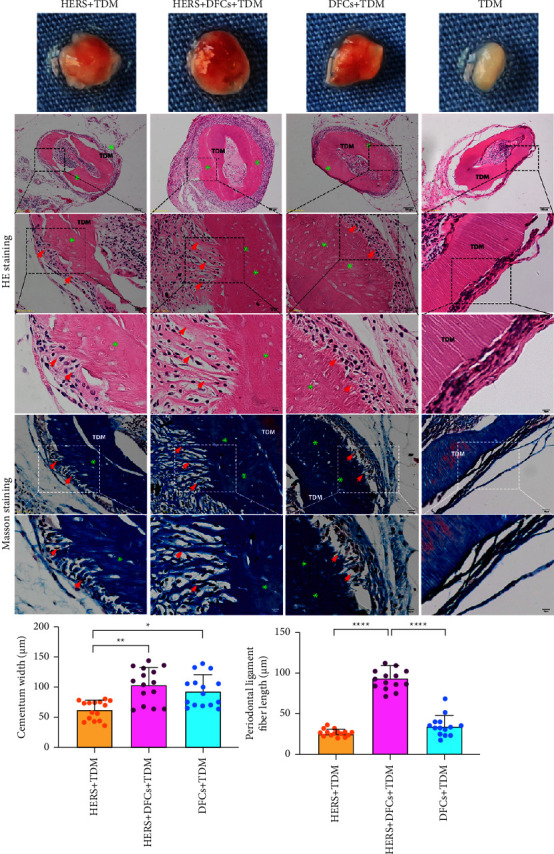
Representative specimen harvested from greater omentum transplantation and histological staining. The specimen observation showed that the vascular net and soft tissue around the TDM were the most abundant in the HERS + DFCs group. The H&E and Masson staining showed that, except for the blank group (TDM only), a layer of cementum-like structure (indicated by green asterisk) was formed around each TDM, in the surface of which there were periodontal ligament-like fibrous (indicated by red arrows) attachments. The width of the formed cementum-like structure in the HERS + DFCs group was larger than that of the HERS and the DFCs groups. Meanwhile, the periodontal ligament-like fibers were the longest and orderly arranged in the HERS + DFCs group than that of the HERS and the DFCs groups (^*∗*^*p* < 0.05, ^*∗∗*^*p* < 0.01, and ^*∗∗∗∗*^*p* < 0.0001).

There was no adverse reaction, i.e., infection, in any group after TDM *in vivo* transplantation. All specimens were preserved in the greater omentum, and the surfaces of the specimens were covered by fibrous tissues with a vascular network, as shown in Figure 5. The HERS + DFCs group showed the richest vascular network and fibrous tissue coverage, while the TDM group without cell seeding showed the least. As shown in Figure 5, 1/3 of the samples in both HERS and DFCs groups and 2/3 of the samples in HERS + DFC group showed a layer of cementum-like structure around each TDM with fibrous attachment on its surface similar to the periodontal ligament. There was a boundary between the TDM and the newly formed cementum-like structure. The periodontal ligament-like fiber attached to the cementum-like structure. From the histological measurement of the cementum width and the periodontal ligament fiber length, HERS + DFCs group showed the greatest increment in cementum width and periodontal ligament fiber, followed by DFCs and HERS group. In addition, the cementum- and periodontal ligament-like structures in the HERS + DFCs group were most abundant and well arranged. The fiber bundles were thick and long and embedded into the newly formed cementum to form a typical cementum-periodontal ligament-like complex. However, in the blank group, only a fibrous capsule was formed on the surface of the TDM.

**Figure 6:**
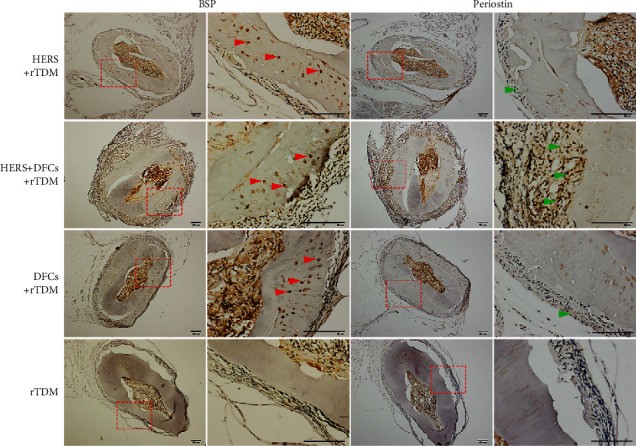
Immunohistochemical staining of TDMs transplanted into the greater omentum. BSP was positively expressed in the newly formed cementum-like structures in each cell-seeded group, especially in the lacunae (indicated by red arrows), while periostin was positively expressed in the newly formed periodontal ligament-like structure (indicated by green arrows), especially in the HERS + DFCs group. In the blank group (TDM only), there was no new tissue formation or attachment on the surface of TDM and the expression of BSP and periostin was negative.

The results of immunohistochemical staining for anti-BSP and antiperiostin are shown in Figure 6. BSP was positively expressed in the newly formed cementum-like structure in each group except for the blank group, especially in the lacunae, while periostin was positively expressed in the newly formed periodontal ligament-like structure, especially in the HERS + DFCs group. In the blank group, no new tissue was attached to the surface of TDM, and BSP and periostin were negatively expressed on the surface of TDM.

## 4. Discussion

The realization of the periodontal regeneration should be based on the developmental principles [5, 6]. The interactions among the dentin, HERS, and DFCs direct the cellular differentiation and periodontal tissue formation [23, 24]. In the present study, the DFCs and HERS cells were seeded individually or in combination onto the surface of TDM to recapitulate the interactions between them during the periodontal formation. The TDMs seeded with and without cells were transplanted into the greater omentum to further explore the roles of HERS cells, DFCs, and dentin matrix in periodontal regeneration. Structures resembling the cementum and the periodontal ligament were found to form on the surface of TDM when DFCs and HERS cells were seeded individually or in combination. Nevertheless, the quantity and quality of the newly formed periodontal structures were different among groups. The group with the combined use of TDM, HERS, and DFCs regenerated the most abundant and typically arranged cementum-periodontal ligament-like structures.

The development and application of bioscaffold materials are important aspects of tissue engineering. Very few scaffold materials have been developed for regenerating intact periodontal tissues. Studies have shown that decellularized dentin matrix could be used to regenerate dental tissues due to its nonimmunogenicity, suitable mechanical properties, and important content of odontogenic protein factors [9, 23, 25]. The dentin matrix contains about 30% organic components, which play a critical role in the development and regeneration of dentin and cementum [26, 27]. Guo et al. combined DFCs with TDM and found TDM a promising biological scaffold for dentin regeneration [9]. In addition, they also found that after the combined transplantation of TDM and DFCs into the alveolar sockets, there were dental pulp and periodontal ligament-like tissues formed [8]. Li et al. have found that human TDM can secrete a variety of protein factors, which will not only promote the proliferation and differentiation of DFCs, but also form a net structure that controls mineralization formation [10]. Yang et al. used the TDM leaching solution to culture DFCs and found a significant increase in the expression of BSP and DMP-1 genes, suggesting that DFCs have an enhanced ability to differentiate into dentin and osteoblast/cementum after induction with TDM-secreted factors. Cementum and periodontal ligament-like complexes were regenerated after subcutaneous transplantation of TDM combined with dental follicle cells in nude mice [28]. These studies suggested that TDM scaffolds not only have a good biocompatibility, but also provide an inductive microenvironment for DFCs to promote their differentiation into cells that produce the periodontal tissues. The present study also concluded that TDM is a suitable scaffold material for the regeneration of periodontium that can effectively induce the differentiation of HERS and DFCs into cementoblasts and periodontal ligament fibroblasts.

In tissue engineering, adequate amounts of seed cells, satisfactory cell viability, and the arrangement of cells on the scaffold material are beneficial to the regeneration of the target tissue; otherwise, the quality and quantity of regeneration will be diminished [29, 30]. It has been suggested that when cells were enzymatically digested, intercellular connections were disrupted, extracellular matrix and proteins were lost, and cell viability was decreased [31, 32]. Therefore, in the present study, in order to improve the quantity and quality of the transplanted cells, we seeded the cells with a high density and then cultured them *in vitro* for a short period of time to rebuild the cell-to-cell connections and ensure a better cell vitality and attachment to the TDM. SEM examination also showed that both HERS cells and DFCs grew well on the surface of TDM. Therefore, the short-term culture *in vitro* is beneficial for improving the further functional differentiation of the seed cells after transplantation *in vivo*.

Another concern about the seed cells is that the cellular behavior and the availability of cells from different developmental stages are different. The HERS is formed after the completion of crown formation at the end of the bell stage of tooth germ development. It has been suggested that HERS cells are a group of pivotal epithelial stem cells that function as the regulation center and play crucial roles in directing the root development as well as the periodontal formation. HERS cells exhibit multiple differentiation potential and mineralization ability and have the capacity to differentiate into cementoblasts and periodontal fibroblasts, participating in the direct formation of the periodontal structures through epithelial-mesenchymal transition [33, 34]. After the root formation is completed, HERS cells were fenestrated by DFCs and reduced to epithelial rests of Malassez (ERM). As the descendants of HERS, ERM disperses in the adult periodontal ligament space and has also been found to possess stem cell properties [35] and play significant roles in periodontal homeostasis and repair [36, 37]. ERM cells have been suggested as the seed cells for periodontal regeneration and could be obtained in adults, while HERS cells could only be isolated from the developing tooth germs. Nevertheless, these ERM cells are the terminal descendants of HERS, which may lose some of the original characteristics of HERS cells. What is more, immortalized HERS cell lines have been established that representing the key features of primary HERS cells for tooth regeneration, thereby providing an unlimited cell source for substitutes of HERS cells [38]. Unlike the development change of HERS and ERM, the DFCs act as the precursors which will differentiate into cementoblasts, periodontal ligament cells and osteoblasts that give rise to cementum, periodontal ligament, and alveolar bone, respectively. Among the descendants of DFCs, the periodontal ligament cells (PDLCs) have been found to exhibit stem cell properties and suggested as the promising seed cells for periodontal regeneration. The PDLCs could be easily obtained from the adult teeth, usually from the premolars that need orthodontic extractions, while the DFCs could also be easily obtained from the developing wisdom teeth that need extractions for not only orthodontic reasons but also the impaction reasons [39]. Nevertheless, it has been found that DFCs showed stronger periodontal regeneration potential than the PDLCs. The expression of proteins revealed by proteomic analysis also showed evident differences between DFCs and PDLCs [18, 39, 40]. Taken together, based on the cell performance and availability, HERS cells and DFCs would have stronger potential for the application in periodontal regeneration.

HERS plays an indispensable role in the induction and regulation of DFC differentiation during tooth development and regeneration, both in terms of the secreted factors and the direct contact. Previous studies have found that HERS cells can release a variety of protein factors, such as BMP-2, BMP-4, EMPs, and TGF-*β*, to induce the differentiation of DFCs [19]. After being induced by HERS, dental follicle cells can express more cementoblast-related genes and proteins, and there were cementum-like and periodontal ligament-like tissues formed after transplanting the DFCs *in vivo* [16]. Some studies found that the induction and regulation of tooth development and regeneration were also influenced by the adjacent relationship between HERS and DFCs. In addition to paracrine, direct intercellular contact between HERS and dental follicle cells as well as changes in extracellular matrix compositions mediated the epithelial-mesenchymal interaction [41, 42].

As previously mentioned, the periodontal regeneration with the combined use of TDM and DFCs depended on the microenvironment. Although the combined use of TDM and DFCs can regenerate periodontal tissues successfully when transplanted into fresh extraction alveolar sockets, the quality and quantity of the regenerated tissues would be compromised when transplanted into the healed alveolar bone and other nondental microenvironments [8, 11, 12, 43]. The cellular composition of the different transplantation microenvironments varied. In the present study, the greater omentum was selected as the transplantation site. For one thing, the omental pouch site could not only provide a large enough space to accommodate the grafts but also have a rich vascular network and could promote the neovascularization in ischemic tissues [9, 44]. For another, the cell composition and structure of the greater omentum are relatively simple and are immunoprotective for the grafted cells or tissues [45]. The greater omentum has no hard tissues and mineralization-relevant cells, which makes it a suitable environment for studying the cell differentiation and interaction of odontogenic cells. Regarding the alveolar sockets, the cell composition and the structure are complex, which contain hard tissues and mineralization-relevant cells. These factors could exert significant impacts and make it difficult to demonstrate the differentiation and interaction of the target cells or tissues. As revealed in the present study, complete cementum-periodontal ligament-like structures were regenerated in greater omentum transplantation. More importantly, the quality and quantity of the regenerated structures with the combined use of TDM, DFCs, and HERS were superior to those with TDM and DFCs or HERS only. This indicated that the combined interactions among TDM, HERS, and DFCs are essential to stimulating the differentiation of periodontium-forming cells and therefore the formation of the periodontal structures in the nondental microenvironments.

The study was not without its limitations. One of the limitations is that the greater omentum was selected as the transplantation site mainly because of its relatively simple cell composition in the present study. It is beneficial for the evaluation of the role and the interaction of TDM, DFCs, and HERS cells during the periodontal formation. Nevertheless, for better mimic the restoration of periodontal tissues for future clinical application, periodontal defect models or transplantation in the alveolar sockets are expected in the future studies. Another limitation is that the combinatorial complex *in vitro* was cultured only for 4 days at most and was examined with SEM only. To better evaluate the cell growth, differentiation, and interaction under the induction of the TDM, a longer culture period *in vitro* and other examining approaches could be performed after dimineralization and sectioning of the TDM complex, such as the immunofluorescence staining, immunohistochemistry, and the H&E and Masson stains.

## 5. Conclusions

In the present study, HERS cells and DFCs were seeded on the surface of TDM individually or in combination and then transplanted into the greater omentum to explore their interactions and the potential for periodontal regeneration from the perspective of epithelial-mesenchymal interactions. With the limitations of the present study, the results showed that TDM served as an effective scaffold material to promote periodontal regeneration by inducing HERS and DFC interaction and differentiation towards cementoblasts and periodontal ligament fibroblasts. Abundant and typical cementum and periodontal ligament-like structures have been successfully regenerated in the group with the combined use of TDM, DFCs, and HERS cells, indicating that it could be an effective and promising strategy for periodontal regeneration, especially in the nondental microenvironments.

## Figures and Tables

**Table 1:** Groups of cell seeding.

Groups	1	2	3	4
Cell	HERS	HERS + DFCs	DFCs	Blank
Number of samples	3	3	3	3
Medium	EpiCM + *α*-MEM (1 : 1)			
Material	TDM			

## Data Availability

All data generated and/or analyzed during the current study are available from the corresponding authors on reasonable request.
